# Continuous Phase Plate Structuring by Multi-Aperture Atmospheric Pressure Plasma Processing

**DOI:** 10.3390/mi10040260

**Published:** 2019-04-18

**Authors:** Duo Li, Na Li, Xing Su, Kan Liu, Peng Ji, Bo Wang

**Affiliations:** Center for Precision Engineering, Harbin Institute of Technology, Harbin 150001, China; suxinghit@126.com (X.S.); liukan@stu.hit.edu.cn (K.L.); jeffersonhit@126.com (P.J.)

**Keywords:** atmospheric pressure plasma processing, continuous phase plate, optic figuring, multi-aperture processing

## Abstract

A multi-aperture atmospheric pressure plasma processing (APPP) method was proposed to structure the continuous phase plate (CPP). The APPP system was presented and removal investigation showed the removal function of APPP was of a high repeatability and robustness to the small disturbance of gas flows. A mathematical model for the multi-aperture structuring process was established and the simulation analysis indicated the advantages of the proposed method in terms of balancing the efficiency and accuracy of the process. The experimental results showed that multi-aperture APPP has the ability to structure a 30 mm × 30 mm CPP with the accuracy of 163.4 nm peak to valley (PV) and 31.7 nm root mean square (RMS).

## 1. Introduction

Continuous phase plates (CPPs) are essential diffractive optical elements in the light path of laser-driven inertial confinement fusion (ICF) systems, such as the National Ignition Facility [1], Laser Megajoule [2] and the SG-III laser facility [3]. The continuously varying structured topography of CPPs can modulate the incident laser to realize beam shaping and smoothing, and thus achieve the uniform illumination of the target surface [4]. The complexity of surface topography (with small spatial periods and large surface gradients) makes it difficult to fabricate/structure CPPs with high efficiency and accuracy. Magnetorheological finishing (MRF) has been used to fabricate large-aperture CPPs [5], in which the spatial periods of microstructures are usually larger than 4 mm, and the peak to valley (PV) of the structure height is as large as several microns. Smaller structures on CPPs are difficult for MRF due to the limitation of tool sizes. Ion beam figuring (IBF) has the potential to figure structures down to 1 mm and different sizes of removal spots can be achieved with a shielding diaphragm. However, the low removal rate limits its application to large and steep CPPs [6].

Atmospheric pressure plasma processing (APPP) is a promising technique for the modification, decontamination, and etching of polymers and glasses [7,8,9,10,11,12,13]. Recently, APPP has received a great deal of interest in optical fabrication because of its deterministic high material removal rate, controlled millimeter tool spot, and no subsurface damage. It is based on pure chemical reactions between the surface atoms of silicon-based materials and reactive fluorine radicals generated by the plasma at atmospheric pressure, which avoids the introduction of damage to the processed surface and significantly lowers the processing cost. Jourdain et al. [14] adopted the reactive atom plasma process for the figuring of large telescope optics. An inductively coupled type plasma torch with a De-Laval nozzle was used to generate a Gaussian removal footprint. For the management of heat transfer, an adapted tool-path strategy was combined with an iterative figuring procedure. Due to the high thermal nonlinear effect of inductively coupled plasma, Dai et al. [15] developed an algorithm based on the nested pulsed iterative method to compensate for this time-varying non-linearity by varying the dwell time. With the compensated dwell time, the surface error converged from 4.556 λ PV (peak-to-valley) to 0.839 λ PV in one iterative figuring. More commonly, capacitively coupled plasma is adopted for high precision processing applications. Meister and Arnold [16] investigated the atmospheric plasma jet machining of fused silica. A three-dimensional finite element heat transfer model was built to consider spatio-temporal variations of the surface temperature and temperature-dependent material removal. The figuring convergence was improved by an iterative correction of the targeted removal according to the modelling results. Sun et al. [17] investigated the etching characteristics of plasma chemical vaporization for reaction-sintered SiC by optimizing the gas composition. Experiments showed that a large surface roughness resulted from the different etching rates of the different components in SiC. By applying the optimum gas composition, a smooth surface was obtained after plasma etching if the etching rate of the Si component was equal to that of the SiC component. Deng et al. [18] combined plasma chemical vaporization machining and plasma-assisted polishing. Plasma chemical vaporization machining was performed to remove the subsurface damage layer, while plasma-assisted polishing (including plasma modification and soft abrasive polishing), was performed for damage-free surface finishing. The results indicated that a flat and scratch-free surface with a root mean square (RMS) roughness of 0.6 nm was obtained.

The stable and controllable Gaussian-shape removal function makes APPP possible to fabricate structured surfaces with high accuracy and efficiency. Also, multi-aperture processing (with multiple-scale removal functions) is enabled to target different scales of surface features to increase the overall machining efficiency. However, little research has been reported on structuring CPPs with the multi-aperture APPP technique. In this paper, a multi-aperture APPP method was proposed for CPP structuring. The APPP system was first introduced, and its removal characteristics were investigated in two aspects: the repeatability and robustness. Then, a mathematical model for multi-aperture APPP dwell time solution was established and simulation analysis was performed to study the structuring efficiency and accuracy. Finally, the experimental processing was carried out to validate the effectiveness of the proposed multi-aperture APPP to structure CPPs.

## 2. APPP System Configuration and Removal Investigation

### 2.1. System Configuration

**Figure 1:**
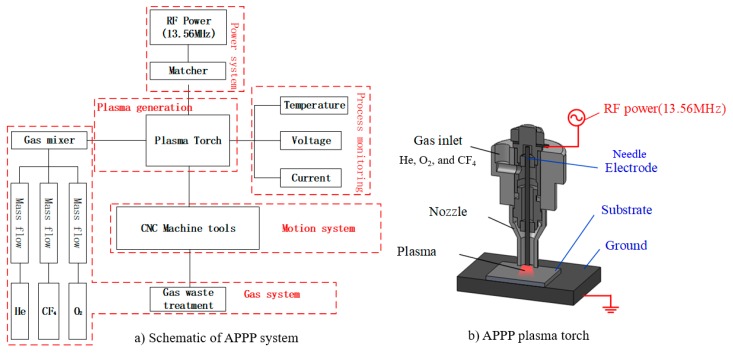
Schematic diagram of the atmospheric pressure plasma processing (APPP) system, reproduced with permission from [19].

A schematic diagram of the APPP setup is shown in Figure 1 [19]. The setup included a plasma generation torch (capacitively coupled), a gas supply module, and a multi-axis computer numerical control machine tool. A 13.56 MHz radio frequency (RF) power was applied to the central aluminum needle as a positive electrode and the workbench was grounded. Thus, the substrate (fused silica) placed on the workbench served as a dielectric barrier layer. In addition, a ceramic nozzle coaxial with the needle electrode was used to restrain the gas flow. The inner mixed gas, including He, O_2_, and CF_4_, was excited in the RF electromagnetic field. The flow rates of gases were controlled by the multichannel mass flow controller.

The plasma (He and O_2_) generated by radio frequency power was regarded as a chemical reactor; the reactant gas (CF_4_) fed into the reactor was decomposed by the collision of plasma electrons into active species [20]. These reactive radicals (F) which were carried by plasma jet flow, diffused to the substrate and reacted with the CPP surface material (SiO_2_) to accomplish the nanometric removal process. The balanced chemical reaction equation can be described as ${\mathrm{SiO}}_{2}{+\mathrm{CF}}_{4}\to {\mathrm{SiF}}_{4}↑{+\mathrm{CO}}_{2}↑$.

### 2.2. Removal Investigation

**Figure 2:**
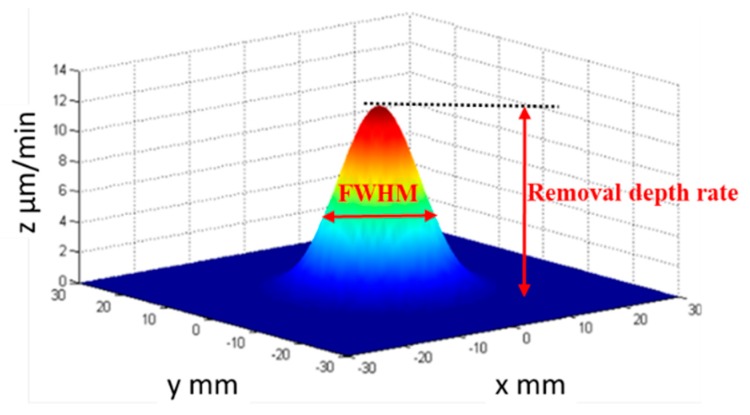
Gaussian shape removal function of APPP.

APPP is a sub-aperture deterministic computer-controlled optical surfacing (CCOS) method and its removal characteristics must be investigated before the fabrication process. Figure 2 shows its typical removal function, which is a Gaussian shape. The Gaussian shape of the removal function is favorable for sub-aperture figuring techniques to correct the optical form error [21]. The removal depth rate and the full width at half maximum (FWHM) are generally used to characterize the removal function, as shown in Figure 2. As the size of the needle electrode is changeable, controllable FWHMs can be easily achieved in the APPP, which is beneficial for structuring the surface topography with different spatial periods. 

In the actual CPP structuring process, the removal function repeatability and its robustness to disturbances were essential, which determined the process convergence and structuring accuracy. APPP removal experiments needed to be carried out. The plasma source was stabilized before the experimental processing. The sample material was fused silica and process parameters are listed in Table 1. 

#### 2.2.1. Repeatability of the Removal Function

**Figure 3:**
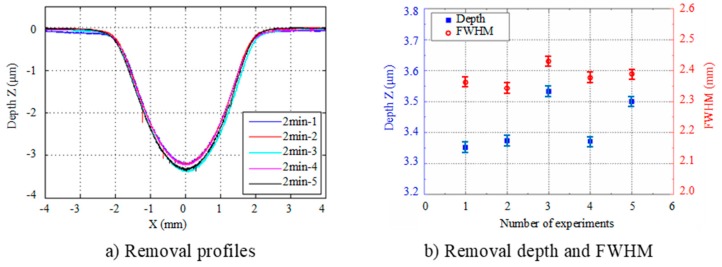
Repeatability of the APPP removal function.

In order to investigate the repeatability of the removal function, five static removal spots were etched using the same process parameters shown in Table 1. The processing time of each spot was 2 min and the removal contour was measured by a stylus profilometer (PGI 1240, Taylor Hobson, Leicester, UK). The repeatability results are shown in Figure 3.

From the results, it can be seen that the maximum deviation of removal depth was about 5%, and the maximum deviation in FWHM was about 3.6%. The Gaussian removal function repeatability of APPP met the requirements for structuring CPPs. 

#### 2.2.2. Robustness of the Removal Function to Gas Flow Disturbance

**Figure 4:**
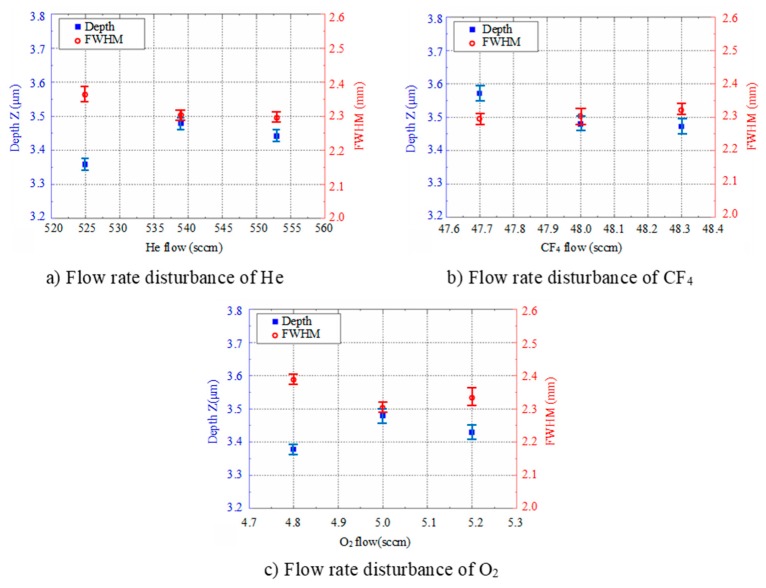
Experiment results for small disturbance robustness.

In actual processing, small disturbances in gas flow are unavoidable. Thus, it is necessary to study the robustness of the removal function under gas flow disturbances. The experimental parameters shown in Table 2 were used and other parameters were kept the same as in Table 1. The measurement and characterization results are shown in Figure 4.

According to the results, for the small disturbance of He, CF_4_ and O_2_ flow, the maximum deviation in the depth direction was about 3.5%, 2.8% and 2.9%, respectively, and the maximum deviation of the FWHM was about 2.9%, 1.2% and 3.6%. This was in the same range as the results from the repeatability experiments, indicating that the removal function of APPP was robust to the small disturbance of gas flows.

## 3. Multi-Aperture Structuring Principle and Analysis

**Figure 5:**
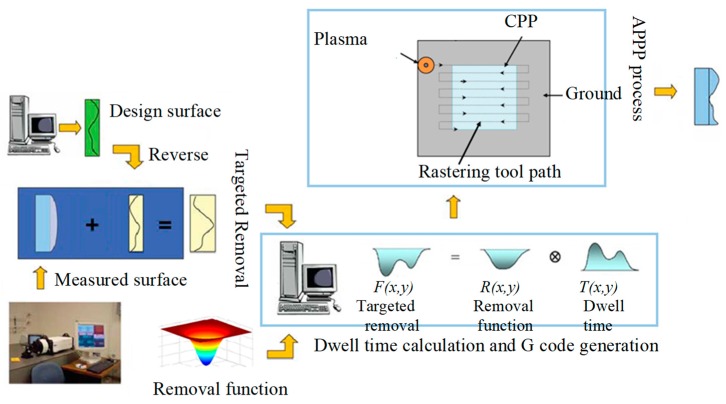
APPP structuring process flow chart.

The APPP structuring process flow chart is illustrated in Figure 5. First, to obtain the targeted removal map, the design CPP surface height data was inverted and superimposed with the initial substrate surface (measured by interferometer). Then, according to the removal map and selected removal functions, the dwell time was calculated (as a deconvolution optimization process), and the corresponding numerical control code was generated for the machine tool. Finally, the APPP process was performed to structure the phase topography on the optic substrate.

Due to the complexity of CPP topography, the selection of the removal functions is essential to also meet the structuring capacity and processing efficiency. APPP removal functions with small FWHMs are suitable for structuring topography with small spatial periods. While the processing time might be unaffordable, removal functions with large FWHMs are associated with higher removal rates, but their structuring capacity is limited. 

For the conventional CCOS dwell time calculation, only a single removal function is considered in each optimization process. Taking both the CPP structuring efficiency and accuracy into account, this study proposed a multi-aperture optimized structuring method, where multiple removal functions were considered, and the corresponding dwell time was simultaneously solved in one optimization process. The mathematical model and the simulation analysis are presented in the following section.

### 3.1. Mathematical Model

For conventional CCOS (using a single removal function), the target removal is equal to the convolution between the single removal function and the dwell time [22]. In the multi-aperture structuring process, the total target removal amount is the sum of convolutions between multiple removal functions and the corresponding dwell time. It can be expressed by the mathematical model shown by the following equation,

(1) $$F(x,y)=R_{1}(x,y)⊗T_{1}(x,y)+R_{2}(x,y)⊗T_{2}(x,y)+\cdots +R_{n}(x,y)⊗T_{n}(x,y)$$

where

F(x,y)—target removal;

$$R_{i}(x,y)\text{—}i\mathrm{th}\;\mathrm{removal}\;\mathrm{function};$$

$$T_{i}(x,y)\text{—}i\mathrm{th}\;\mathrm{corresponding}\;\mathrm{dwell}\;\mathrm{time}.$$

**Figure 6:**
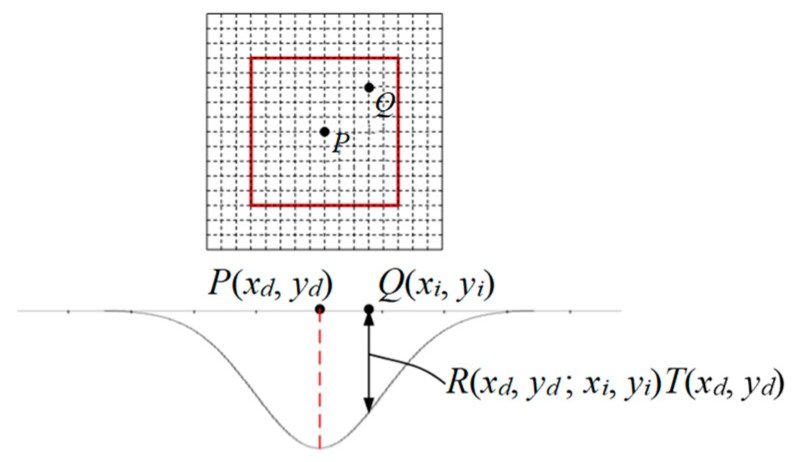
Schematic of the removal amount on dwell points.

The Fourier transform method and the conventional deconvolution method used in the CCOS are be suitable to solve the problem in Equation (1). Therefore, a matrix-based optimization model was established to obtain the dwell time solution for multiple removal functions. As shown in Figure 6, for a removal function, when the plasma torch resides at a certain dwell point *(x_d_,y_d_)*, the amount of removal at any point *Q(x_i_,y_i_)* can be determined by the Equation (2).

(2) $$F(x_{i},y_{i})=R(x_{d},y_{d};x_{i},y_{i})T(x_{d},y_{d})$$

Assuming that the number of removal points is N, and the number of resident points is M, then the removal amount of all the dwell points to the point is as shown in Equation (3).

(3) $$F(x_{i},y_{i})=\sum_{j}^{M}R(x_{j},y_{j};x_{i},y_{i})T(x_{j},y_{j})$$

Take two different removal functions for the multi-aperture structuring process as an example. Assuming that the number of dwelling points of the first removal function is *M*_1_, and the number of dwelling points of the second removal function is *M*_2_, Equation (1) can be converted into the matrix-based form as follows,

(4) $$\begin{array}{l} \left[\begin{array}{c} f_{1}\\ f_{2}\\ \vdots \\ f_{N} \end{array}\right]=\left[\begin{array}{cccc} r_{11}^{\prime } & r_{12}^{\prime } & \cdots & r_{1M_{1}}^{\prime }\\ r_{21}^{\prime } & r_{22}^{\prime } & \cdots & r_{2M_{1}}^{\prime }\\ \vdots & \vdots & \vdots & \vdots \\ r_{N1}^{\prime } & r_{N2}^{\prime } & \cdots & r_{NM_{1}}^{\prime } \end{array}\right]\left[\begin{array}{c} t_{1}^{\prime }\\ t_{2}^{\prime }\\ \vdots \\ t_{M_{1}}^{\prime } \end{array}\right]+\left[\begin{array}{cccc} r_{11}^{\prime \prime } & r_{12}^{\prime \prime } & \cdots & r_{1M_{2}}^{\prime \prime }\\ r_{21}^{\prime \prime } & r_{22}^{\prime \prime } & \cdots & r_{2M_{2}}^{\prime \prime }\\ \vdots & \vdots & \vdots & \vdots \\ r_{N1}^{\prime \prime } & r_{N2}^{\prime \prime } & \cdots & r_{NM_{2}}^{\prime \prime } \end{array}\right]\left[\begin{array}{c} t_{1}^{\prime \prime }\\ t_{2}^{\prime \prime }\\ \vdots \\ t_{M_{2}}^{\prime \prime } \end{array}\right]\\ =\left[\begin{array}{cccc} r_{11}^{\prime } & r_{12}^{\prime } & \cdots & r_{1M_{1}}^{\prime }\\ r_{21}^{\prime } & r_{22}^{\prime } & \cdots & r_{2M_{1}}^{\prime }\\ \vdots & \vdots & \vdots & \vdots \\ r_{N1}^{\prime } & r_{N2}^{\prime } & \cdots & r_{NM_{1}}^{\prime } \end{array}\begin{array}{cccc} r_{11}^{\prime \prime } & r_{12}^{\prime \prime } & \cdots & r_{1M_{2}}^{\prime \prime }\\ r_{21}^{\prime \prime } & r_{22}^{\prime \prime } & \cdots & r_{2M_{2}}^{\prime \prime }\\ \vdots & \vdots & \vdots & \vdots \\ r_{N1}^{\prime \prime } & r_{N2}^{\prime \prime } & \cdots & r_{NM_{2}}^{\prime \prime } \end{array}\right]\left[\begin{array}{c} \begin{array}{c} t_{1}^{\prime }\\ t_{2}^{\prime }\\ \vdots \\ t_{M_{1}}^{\prime } \end{array}\\ \begin{array}{c} t_{1}^{\prime \prime }\\ t_{2}^{\prime \prime }\\ \vdots \\ t_{M_{2}}^{\prime \prime } \end{array} \end{array}\right] \end{array}$$

The solution of the linear equations shown in Equation (4) is the dwell time of the two APPP removal functions involved in the multi-aperture structuring. As the sum of the number of dwell points is larger than the number of removal points (*M*_1_ + *M*_2_ > *N*), the optimal solution of the overdetermined equations is generally needed. According to the two conditions of the minimum of the residue error and the non-negative dwell time, the optimization objective and constraints of the linear equations are as the follows,

(5) $$\begin{array}{l} \underset{t}{\min}g(t)=\frac{1}{2}{\left\|Rt-f\right\|}_{2}^{2}\\ s.t.\;t\geq 0 \end{array}$$

where ${\left\|Rt-f\right\|}_{2}$ is the 2-norm of the residual error. The linear equations in Equation (4) can be solved using the constrained optimization method [23].

### 3.2. Simulation and Analysis

**Figure 7:**
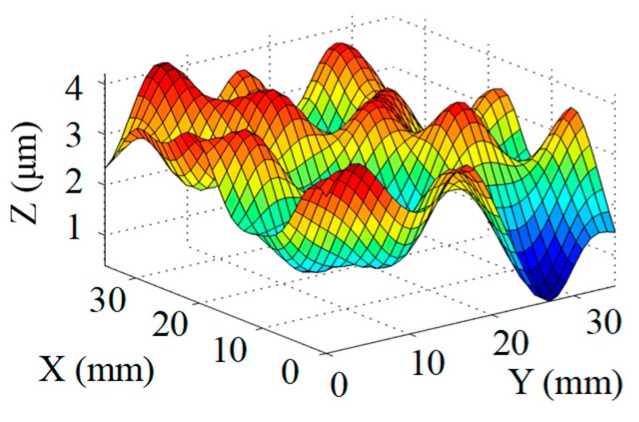
CPP surface for simulation analysis.

Structuring simulation was performed to validate the proposed multi-aperture method. A 36 mm × 36 mm CPP surface was used (as shown in Figure 7) and two experimentally obtained removal functions with different FWHMs (as shown in Table 5 and Table 6) were adopted. Structuring simulation with single removal functions and multiple removal functions was performed and compared. The simulation results are shown in Table 3 and Table 4. 

It can be seen from the simulation results that when the single removal function ① (with a larger FWHM and higher removal rate) was used, the total processing time was 5.61 min, but the residue error was high, as the calculated PV value and RMS value were 0.46 μm and 0.055 μm, respectively. When the removal function ② (with a smaller FWHM and a lower removal rate) was used, the residue error was almost zero (the PV and RMS values of the error reached 7.55 × 10^−15^ μm and 8.05 × 10^−16^ μm, respectively), but the structuring efficiency was very low, as the processing time was 340.94 min. When the two removal functions were adopted for combined multi-aperture process, the total processing time was 57.24 min (where the removal function ① and the removal function ② occupied 4.64 min and 52.6 min, respectively). The PV and RMS values of the residue error were 1.49 × 10^−5^ μm and 1.11 × 10^−6^ μm, respectively, showing the advantages of both processing efficiency and accuracy.

## 4. Experiments and Results

**Figure 8:**
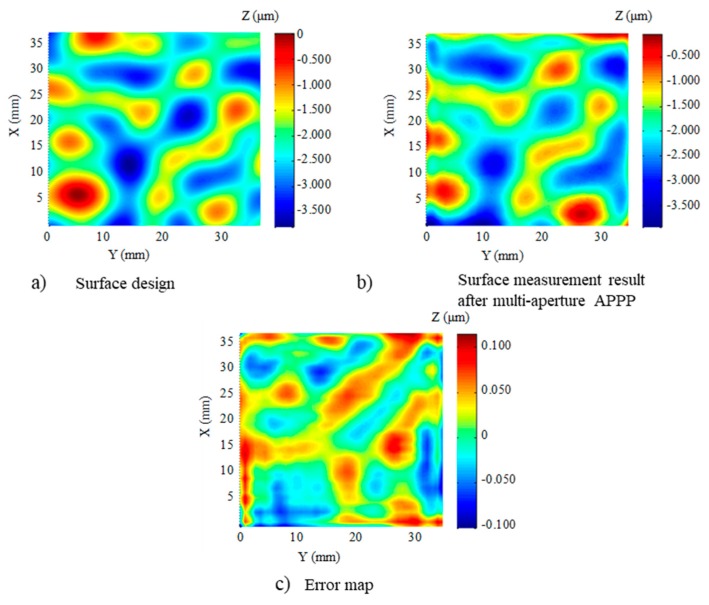
Multi-aperture APPP results of CPP.

According to the preliminary experiments, two removal functions were experimentally obtained using Φ3 mm (with a removal rate of 20.8 μm/min and FWHM 5.7 mm) and Φ1 mm (with a removal rate of 1.65 μm/min and FWHM 2.4 mm) diameter electrodes. Other process parameters were adopted to achieve the stable plasma etching process, as listed in Table 5 and Table 6. The surface design is shown in Figure 8a. Based on the simulation results and calculated dwell time, the CPP structuring was performed using multi-aperture APPP with two removal functions. 

The surface figure was then measured by a phase shifting interferometer (Zygo Corp., Berwyn, PA, USA). The structuring result is shown in Figure 8b. Compared with the design surface, the processing error map is shown in Figure 8c. It shows that the processing error in the range of in the central CPP (30 mm × 30 mm) was 163.4 nm PV, which accounts for 4.4 % of the total PV value of the surface structures. The RMS value of the error was 31.7 nm. This result is comparable to the processing accuracy obtained by magnetorheological polishing [4]. The experimental results indicate the potential of multi-aperture APPP to structure CPPs with high accuracy and efficiency.

## 5. Conclusions

In order to structure CPPs with high efficiency and accuracy, a multi-aperture APPP method was investigated with stable and controllable Gaussian-shape removal functions. The APPP system and removal investigation were introduced. The APPP removal function was of high repeatability and robustness to the small disturbance of gas flows. A multi-aperture mathematical model for the dwell time solution was established and the simulation analysis indicated the advantages of the proposed method in terms of balancing the process efficiency and accuracy. The experimental results showed the successful fabrication of a 30 mm × 30 mm CPP with an accuracy of 163.4 nm PV and 31.7 nm RMS, which demonstrates the potential of the multi-aperture APPP to structure complex CPPs.

## Figures and Tables

**Table 1:** Process parameters for removal investigation.

He Flow (sccm)	CF_4_ Flow (sccm)	O_2_ Flow (sccm)	Distance (mm)	Power (W)
539	48	5	3	48

**Table 2:** Process parameters for small disturbance robustness.

Parameters	Nominal Value	Disturbance Value
He flow (sccm)	539	525, 539, 553
CF_4_ flow (sccm)	48	47.7, 48, 48.3
O_2_ flow (sccm)	5	4.8, 5, 5.2

**Table 3:** Simulation results of dwell time and residue error distribution.

Machining Mode	Dwell Time Distribution	Residue Error Distribution
Single Removal Function ①	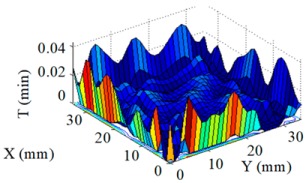	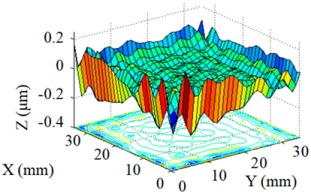
Single Removal Function ②	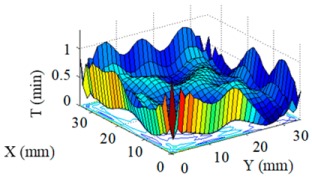	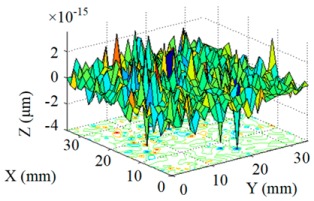
Combined Removal Functions ① and ②	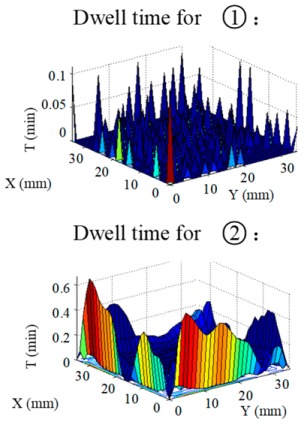	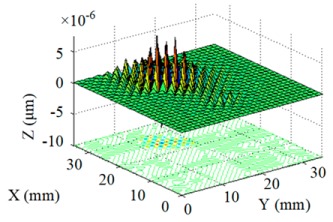

**Table 4:** Characterization of dwell time and residue error.

Machining Mode	Dwell Time (min)	Residue Error	
		PV (μm)	RMS (μm)
Single Removal Function ①	5.61	0.46	0.055
Single Removal Function ②	340.94	7.55 × 10^−15^	8.05 × 10^−16^
Combined Removal Functions ① and ②	57.24 (①/②: 4.64/52.6)	1.49 × 10^−5^	1.11 × 10^−6^

**Table 5:** Process parameters for removal function ①.

Electrode Diameter (mm)	He flow (sccm)	CF_4_ Flow (sccm)	O_2_ Flow (sccm)	Discharge Distance (mm)	Power (W)	Removal Rate (μm/min)	FWHM (mm)
3	539	48	5	3	106	20.8	5.7

**Table 6:** Process parameters for removal function ②.

Electrode Diameter (mm)	He flow (sccm)	CF_4_ Flow (sccm)	O_2_ Flow (sccm)	Discharge Distance (mm)	Power (W)	Removal Rate (μm/min)	FWHM (mm)
1	539	48	5	3	48	1.65	2.4
